# Random forest and Shapley Additive exPlanations predict oxytocin targeted effects on brain functional networks involved in salience and sensorimotor processing, in a randomized clinical trial in autism

**DOI:** 10.1038/s41386-025-02095-2

**Published:** 2025-04-02

**Authors:** Elissar Andari, Kaundinya Gopinath, Erin O’Leary, Gabriella A. Caceres, Shota Nishitani, Alicia K. Smith, Opal Ousley, James K. Rilling, Joseph F. Cubells, Larry J. Young

**Affiliations:** 1 Department of Neurosciences and Psychiatry, College of Medicine and Life Sciences, University of Toledo, Toledo, OH USA; 2 Adjunct Faculty in the Department of Psychiatry and Behavioral Sciences, Emory University School of Medicine, Atlanta, GA USA; 3 Center for Translational Social Neuroscience, Emory University School of Medicine, Atlanta, GA USA; 4 Silivio O. Conte Center for Oxytocin and Social Cognition, Emory National Primate Research Center, Emory University, Atlanta, GA 20329 USA; 5 Center for Systems Imaging Core, Department of Radiology and Imaging Sciences, G-131, Health Science Research Building II, Emory University, Atlanta, GA 30322 USA; 6 The Max Harry Weil Institute, Michigan Medicine, University of Michigan, Ann Arbor, MI USA; 7 Department of Psychiatry and Behavioral Sciences, Emory University School of Medicine, Atlanta, GA USA; 8 Gynecology and Obstetrics, Emory University School of Medicine, Atlanta, GA USA; 9 Research Center for Child Mental Development, University of Fukui, Fukui, Japan; 10 Department of Psychology, Emory University, Atlanta, GA USA; 11 Department of Human Genetics and Psychiatry and Behavioral Sciences, Emory University School of Medicine, Atlanta, GA USA

**Keywords:** Limbic system, Human behaviour

## Abstract

Intranasal oxytocin (IN-OXT) has shown some promises in rescuing social deficits in autism spectrum disorder (ASD) as well as some inconsistencies in long-term trials. We conducted a target engagement study to study the precise effects of different doses of IN-OXT on brain resting-state functional connectivity (rsFC) in ASD. We examined the effects of varying doses of IN-OXT (0 IU, 8 IU, 24 IU, 48 IU) on rsFC in a double-blind, placebo-controlled, within-subject design in 30 male adults with ASD and 17 neurotypical controls (NT) receiving placebo. Random forest analysis was used to classify individuals as ASD or NT. Shapely Additive explanations values were calculated to rank brain functional networks by level of contribution to ASD deficits and to evaluate IN-OXT dose effects. The model predicted ASD diagnosis with an AUC of 94%. Hypoconnectivity between salience/empathy and visual networks, and hyperconnectivity between reward and sensorimotor networks and theory of mind networks were among the strongest predictors of ASD deficits. IN-OXT had a dose-dependent effect on rescuing both deficits described above. Overall, 48 IU dose was more effective, and 24 IU dose was more effective in those who have lower DNA OXT receptor methylation and lower severity of clinical symptoms. Higher doses of OXT might be necessary to enhance empathic responses, and ASD individuals with less support needs and with a preserved OXT system might benefit most from OXT treatment. Applying machine learning approaches in OXT research can provide data-driven unbiased results that can inform future clinical trials.

## Introduction

Autism Spectrum Disorder (ASD) refers to a set of neurodevelopmental disorders characterized by a wide range of social deficits that can vary in phenotypic representation from one individual to another. These behavioral deficits are associated with alterations in resting-state functional connectivity (rsFC), including both hypoconnectivity within sensory and salience networks and between default and attentional networks, as well as hyperconnectivity between somatomotor and subcortical areas [1]. There are no current pharmacological treatments for the core social deficits of ASD.

The neuropeptide oxytocin modulates functional connectivity between areas of social brain network [2, 3], increases signal-to-noise ratios to enhance the salience and reinforcing value of social sensory cues [4–6], and has promising acute effects on brain and behavior in ASD [7–9]. However, chronic intranasal oxytocin (IN-OXT) intake resulted in inconsistent findings in ASD [6, 10, 11]. Young children receiving 32 IU of IN-OXT daily for 12 weeks show improvement on caregiver-rated social responsiveness scale [11], whereas children and adolescents receiving 48 IU daily for 24 weeks did not show improvements on the Aberrant Behavior Checklist modified Social Withdrawal subscale [10]. A lack of understanding of the neurobiological effects (i.e., target engagement) of IN-OXT, and a lack of knowledge about receptor saturation, and the resulting effects on social cognition and behavior likely contribute to inconsistences in long-term clinical trials with IN-OXT in ASD subjects. There is a lack of knowledge of the type of variability in inter-individual responses in ASD to different doses of IN-OXT in different scenarios.

Here, we examined the effects of varying doses of IN-OXT (Syntocinon, Novartis, 0 IU (PL), 8 IU, 24 IU, 48 IU) on rsFC in 30 male adults with ASD. We selected adults instead of children given that we are conducting a mechanistic study and rsFC is more stable in adulthood. We selected a narrow age range of early to middle adulthood to reduce age-based variability in rsFC. The choice behind rsFC stems from the fact that it is one of the most reliable brain-based methods that can be repeated in different sessions without a strong habituation effect, and therefore can be used in within-subject design studies. Studying brain target engagement can add biological validity to oxytocin’s action. We compared rsFC of ASD subjects receiving PL to the rsFC of 17 neurotypical control subjects who also received PL. We used random forest and SHapley Additive exPlanations (SHAP) values to first evaluate rsFC between brain networks that are best predictive of ASD deficits when compared to NT, and to second identify brain functional networks most impacted by IN-OXT in a dose-dependent fashion. *We hypothesized that IN-OXT would normalize the functional connectivity between areas involved in social salience or empathy (anterior cingulate cortex (ACC), anterior insula, visual cortex) and reward processing (ventral striatum, orbitofrontal cortex (OFC))*. We also predicted that the drug would rescue the hypoconnectivity deficit between cortical areas and the hyperconnectivity deficit between subcortical and cortical areas. We further adopted precision medicine approaches to identify moderators for the dose-dependent effect of IN-OXT on rsFC between salience, reward and cortical networks, such as *DNA methylation* of the -1121 to −1119 site of MT2 area (CpG5.6 from Andari et al. [12]) *of the oxytocin receptor gene (OXTR)*, which was previously found to be related to social responsiveness deficits in ASD (SRS-2 assessment) and to hyperconnectivity between striatal (reward) and cortical brain areas (vmPFC) [12]; and *clinical features of ASD, as indexed by ADOS-2* (Autism Diagnostic Observation Schedule-Second Edition) total severity scores.

## Materials and methods

### Participants

We recruited 50 adult men with ASD (between the ages of 18 and 45, IQ > 70) from the Emory Autism Center (IRB protocol #63466) between 2017 and 2019. Of these, 32 were recruited to participate in this study (Emory IRB #9455). 10 were not eligible, 8 families were not available or not reachable, or not willing to participate in a drug trial. Visits occurred at Emory University Hospital. One participant withdrew because of MRI-related anxiety symptoms. Another patient’s data were excluded from analysis because of technical problems with the acquisition of fMRI data (*N* = 30 for data analysis). ASD diagnosis was confirmed with Autism Diagnostic Observation Schedule (ADOS) and Autism Diagnostic Interview (ADI). We recruited 19 neurotypical individuals (NT) who were matched for sex, age and IQ. Two participants withdrew due to lack of interest, leading to 17 controls participating in the trial (see supplemental material). The study was conducted after IRB approval (#9455). Participants started testing procedures after reading and signing the informed consent.

### Experimental procedure

Adults with ASD participated in a total of five visits, each separated by  an approximately one-week interval. The trial adopted a double-blind, placebo-controlled, within-subject design. Participants were screened for their eligibility to participate in an MRI scan. Following consent review during the first visit, the nurse practitioner or the study physician (JFC) conducted a brief history and physical exam. All participants were asked to continue taking their medication as usual and to not make any changes during their participation in this oxytocin trial. Out of 27 ASD subjects who reported their medication use, 19 patients (70%) take anti-depressants, SSRIs, or anti-anxiety medication, 5 patients take medication for irritability and ADHD (18%), 5 patients take medication for allergy (18%), 4 patients take medication for sleep (15%), and 3 patients take antipsychotic medication (11%). ASD subjects also had a fMRI training session to habituate to scanner noise and tasks. For the following visits, vitals were assessed before spray intake, and 15 minutes after (see supplemental material). Ten milliliters of blood were collected by venipuncture 5 min prior to and after spray intake (see supplemental material). Blood samples were collected in EDTA-treated tubes and were immediately centrifuged at 4°C and the plasma was collected and stored at -80°C until analysis. Vitals and blood were also taken from NT subjects before and after nasal spray. fMRI scans were performed (resting state) 40 min after receiving the nasal spray. The primary outcome measures consist of studying the effects of IN-OXT on the modulation of brain functional connectivity between socio-emotional brain areas in ASD. Other primary outcomes consist of studying the effects of IN-OXT on brain function in social tasks which are not part of this manuscript. The study is a clinical trial registered on clinicaltrials.gov (NCT03033784) and approved by the FDA (IND) and the Emory IRB.

### Intranasal OXT administration

Subjects with ASD were scheduled to receive either intranasal placebo (PL), 8 IU, 24 IU, or 48 IU Syntocinon (Novartis, Basil, Switzerland, 40 IU/ml, 5 ml each bottle, each puff is 4 IU) during four visits. The local Emory Hospital pharmacy oversaw compounding the placebo solution and dose preparation of the drug. The random allocation sequence was numbered. The PI, nurse, experimenter, and ASD subjects were blind to the content of the bottles. NT subjects visited only once and received placebo. NT and ASD participants were informed that they might be receiving placebo or one of the various doses of Syntocinon so that both groups had similar expectations. (see supplemental material).

### fMRI acquisition and analysis

Each participant took part in an 8-min rsfMRI scan followed by other tasks that will be reported in future publications. Participants were instructed to keep their eyes open and look at a fixation cross presented on a screen in front of them during the resting-state scans. MR images were acquired on a 3 T Siemens Prisma-Fit scanner with a 64-channel receiver array head and neck coil. Blood oxygenation level dependent (BOLD) contrast rsfMRI scans were acquired using a conventional whole-brain EPI sequence with TR/TE/FA = 3000 ms/25 ms/90°; fifty-eight 2.4 mm thick oblique slices; 1.5 mm × 1.5 mm in-plane resolution (see supplemental material).

Group Independent Component Analysis (ICA) was performed, and functional network connectivity (FNC) was analyzed. Initial GICA was performed with automatic estimation of the number of independent components (ICs) using the minimum description length (MDL) criteria. The ICASSO utility of the GIFT software with random initialization and bootstrapping was used to ensure the stability/reliability of the ICA [13]. Whole-brain maps of strength of the artifact-free ICs (expressed as z-scores), as well as corresponding IC time-series, for each subject were generated by back-reconstruction of the group aggregate mixing matrix [13, 14]. We used the Neurosynth database of brain statistical maps [15] to identify the different networks based on 1000 data points from meta-analysis studies (see supplemental material).

### Random forest analysis

We used a random forest model [16] to classify subjects as either NT or ASD based on rsFC and to determine which connectivity patterns most strongly predict ASD diagnosis (see supplemental material). To optimize the predictions, 5-fold cross validation was used to optimize the hyperparameters in the random forest model. This group of NT was then compared to ASD subjects with various levels of treatment. The model performance was assessed using a nested cross-validation technique [17]. To assess model generalization, a second 5-fold cross-validation procedure was used on the cross-validation process (i.e. nested cross validation). Briefly, this uses all the data and splits the data into five separate folds, with four folds being used to train the data and one fold being used in evaluation. With this approach, each test set consists of data that is never used in a corresponding training process.

Kernel-based SHAP values were used to rank the variables (rsFC) for their ability to predict ASD diagnosis and ASD deficits (hypoconnectivity and hyperconnectivity) as compared to NT participants (using placebo data). This method was also used to detect networks that are most impacted by the different doses of oxytocin.

To assess effects of drug, we conducted model comparisons using the SHAP values from four separate models (PL, 8 IU, 24 IU, 48 IU). Model comparisons were conducted between each dose of IN-OXT and PL in terms of SHAP values, and more specifically PL-Dose (in terms of direction). Then, models were compared to NT receiving PL. Red dots in these comparison figures signify ASD participants. Blue dots represent NT participants. Dots that are closer to the right of the y-axis signify that the value of the subtraction (PL-dose) is positive which means that the treatment reduced SHAP importance contribution of that network and that the difference between ASD and NT is shrinking. Dots to the left of the y-axis mean that the difference between groups of ASD and NT increases and that means that the SHAP value is higher when the treatment is given compared to PL.

Each model compared rsFC in ASD participants at a relevant dose to the NT participants receiving PL. The relative SHAP values were used to assess which networks were affected by the intervention.

#### DNA methylation of *OXTR*

The analysis conducted for the saliva samples collected from ASD subjects was described in detail by Andari and colleagues [12]. The same DNA methylation data from the same patients used in this cited paper are used in this present paper but for different analyses.

### Oxytocin peptide measurement in plasma

Blood samples were collected 5 min prior and post intranasal administration. The blood samples were kept on ice for up to 2 h, before being centrifuged at 4°C. The plasma was removed, transferred to a separate tube, and stored at −80 °C. OXT concentrations were measured using an enzyme immunoassay kit (Enzo Life Sciences, Inc.) (see supplemental material).

#### Additional statistical analysis

We also used a mixed regression model to study the correlation between DNA methylation of the *OXTR* gene in -1121 to −1119 site of MT2 area (CpG5.6 from Andari et al. [12]) and the dose-dependent effect on the main networks of interest (salience and reward networks). This DNA methylation site was used as the independent variable in addition to treatment and age. Functional connectivity between ICA-derived brain resting state networks were used as dependent variables. Similar analysis was conducted for the ADOS calibrated severity total scores and OXT concentration. Independent *t*-tests were used to compare ASD and NT groups in the placebo condition on functional connectivity between ICA-derived brain resting state networks. Repeated measures ANOVA was used to study differences between pre-and post IN-OXT administration for the OXT plasma concentration with doses (0IU, 8IU, 24IU, 48IU) and time (pre and post administration) as within-subject factors. Repeated measures ANOVA was also conducted to study the effects of IN-OXT on functional connectivity in key brain networks. Post-hoc tests with Bonferroni corrections were used to study the main effect of dose.

## Results

### Comparison of Resting-state functional connectivity (rs-FC) in ASD and neurotypical controls

For this analysis, both groups received placebo. In the initial group ICA analysis (*n* = 30 ASD with IN-PL and *n* = 17 NT with IN-PL), we identified 12 functional networks that are above noise (Table S1 and Fig. S1, S2). It includes brain regions involved in *social salience/empathy and pain* (IC1: anterior insula (AI), anterior cingulate cortex (ACC), IC4 and IC13: sensorimotor cortex; and IC15: superior temporal sulcus (STS) or theory of mind network); *the reward and default mode networks* (IC2: striatum, IC5: ventral striatum, nucleus accumbens, medial prefrontal cortex (mPFC), ACC, orbitofrontal cortex (OFC), amygdala, IC14: posterior cingulate gyrus (PCC)); *and the visual/face processing networks* (IC9: occipital cortex, fusiform gyrus, IC16: occipital cortex).

**Fig. 1:**
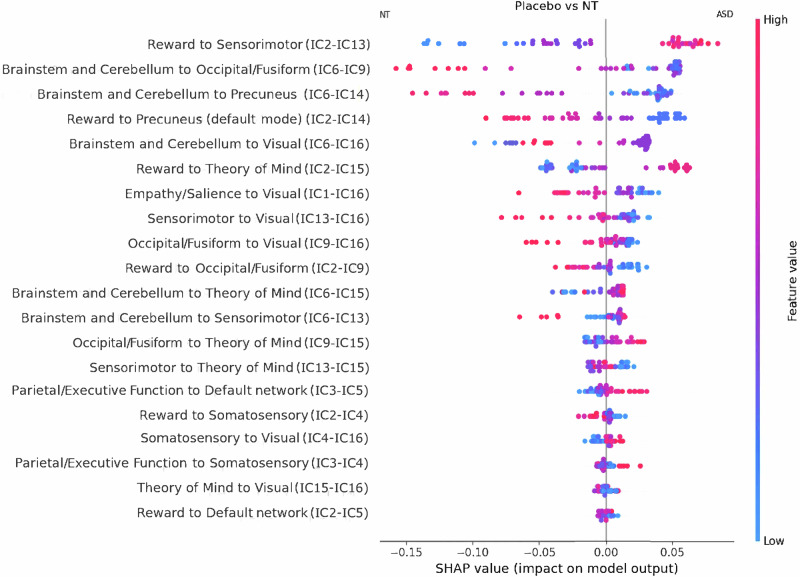
SHAP value of functional connectivity between two given networks ranked in order of importance in distinguishing ASD from NT (both under placebo condition). Pink dots represent a high value (z-score) of the corresponding connectivity between two networks. Blue dots represent a low value of connectivity between the corresponding functional networks. A dot’s horizontal distance from the vertical bar in the middle represents its SHAP value ranging from −0.15 to 0.05. These values represent the overall influence on the prediction for a given subject. Negative SHAP values indicate that the prediction is pushed toward neurotypical controls (NT). Positive SHAP values push the prediction toward ASD. The feature ordering is in descending order of feature importance. The features at the top of the y-axis were more influential in predicting the differences between ASD and NT. This global feature importance is calculated by summing over all subject’s individual SHAP values for a given feature ($f={\sum }_{i\in {subjects}}|{S}_{i}|$ where ${S}_{i}$ is the *i*’th subject’s SHAP value).

The random forest model was able to predict ASD diagnosis or classify individuals as ASD or NT with a 0.944 AUC (Fig. S3). SHAP analysis showed that the hyperconnectivity between reward network (or IC2) and sensorimotor network (IC13) in ASD, in comparison to NT, is the best predictor of diagnosis (Fig. 1). Hyperconnectivity between reward (IC2) and theory of mind network (IC15) (that includes superior temporal sulcus) is also among top contributors (Fig. 1). Also, rsFC between salience/empathy (IC1) and visual networks (IC16) are among the best predictors of ASD deficits (Fig. 1), with ASD individuals characterized by less rsFC between these areas, in comparison to NT participants.

We also compared the two groups on key networks of interest using simple statistical analysis based on our original hypotheses and the results of SHAP value analysis. We found hypoconnectivity between the salience/empathy network (IC1) and the visual network (IC16) (*t* = −2.84, *p* < 0.007) in individuals with ASD in comparison to NT. We also found hyperconnectivity in ASD between the reward network (IC2) and other cortical networks (sensorimotor network (IC13) (t = 2.21, *p* < 0.05), and theory of mind network (IC15) (*t* = 2.42, *p* < 0.02)), in comparison to NT. Exploratory analysis revealed significant reduction in rsFC in ASD between (1) the reward/default network (IC5 which includes default mode mPFC), and the other default mode network (IC14 that includes PCC and precuneus; (*t* = −2.55, *p* < 0.02)), (2) the default mode (IC14) and theory of mind network (IC15) (*t* = 2.69, *p* < 0.02)), and (3) between the theory of mind network (IC15) and visual network (IC16) (*t* = 3.22, *p* < 0.003), in comparison to NT (*p* uncorrected).

**Fig. 2:**
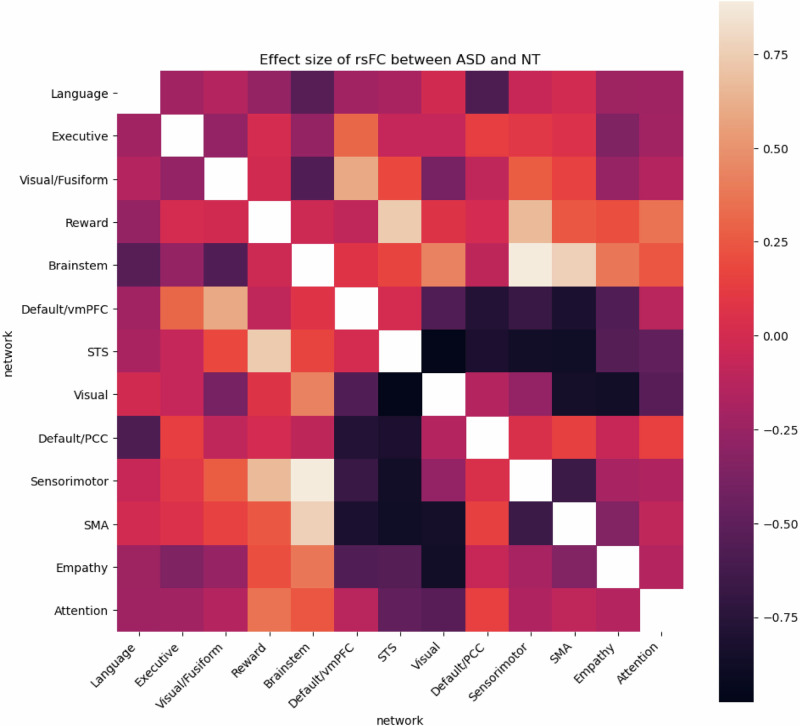
Effect sizes (Cohen’s *d*) of the difference in rsFC between ASD and NT (placebo condition). Darker colors and lighter colors depict stronger differences. Darker colors signify strong effect sizes for ASD to have hypoconnectivity relative to NT while brighter colors signify strong effect sizes for ASD subject to have hyperconnectivity compared to NT.

The effect size map (Fig. 2) revealed that hypoconnectivity between the salience/empathy network (IC1) and visual network (IC16) and between the visual network (IC16) and theory of mind or STS network (IC15), as well as the hyperconnectivity between reward network (IC2) and sensorimotor cortex (IC13); and between reward network (IC2) and theory of mind network (STS, IC15) are among the largest effect sizes in terms of deficits in ASD

### Dose-dependent effects of IN-OXT on rsFC in ASD

**Fig. 3:**
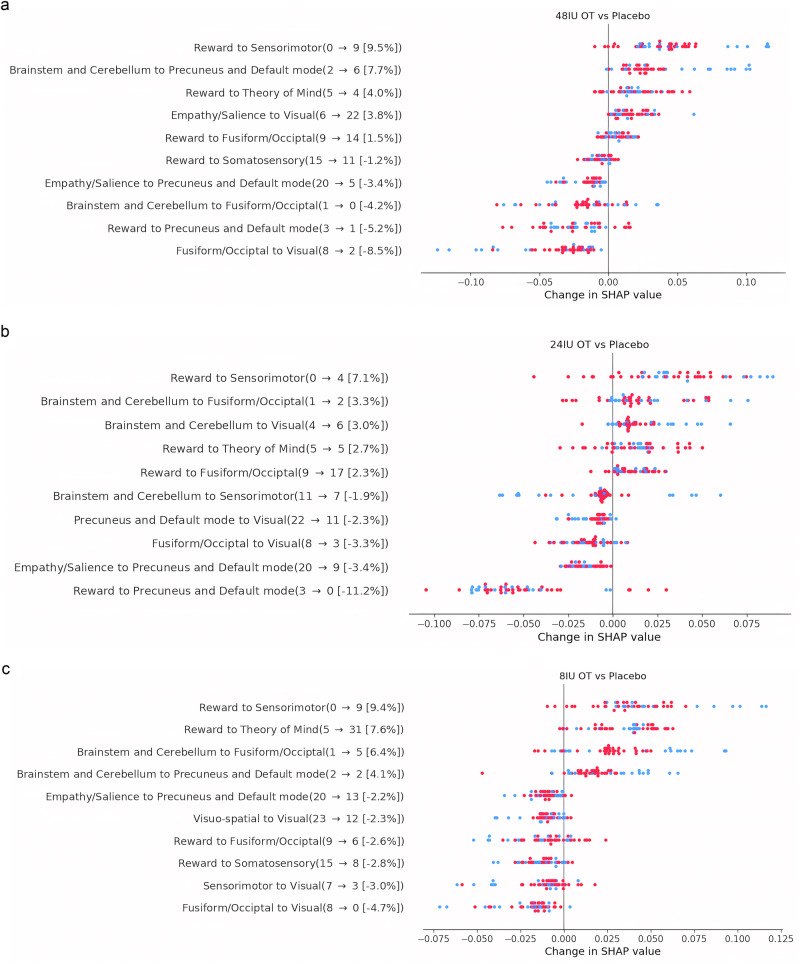
SHAP comparison chart between ASD receiving different doses of IN-OXT and IN-PL. **a** SHAP comparison chart between ASD receiving IN-OXT high dose (48 IU) and ASD receiving IN-PL. Blue and pink dots represent the differences between ASD receiving placebo and ASD receiving 48 IU. The color on these dots represents subject’s diagnosis where pink is for ASD and blue is for NT. Dots that are to the right of the y-axis indicate that the difference between groups is more pronounced in the ASD placebo group (ASD PL – ASD 48 IU). Dots that are to the left of the y-axis indicate that the difference between groups are more pronounced in the ASD 48 IU group, in terms of a particular network contributing more to ASD diagnosis. For instance, for the FC between the reward and sensorimotor cortex, the blue dots to the right with an x-axis value of 0.08 indicates that with 48 IU, the FC between these networks for this subject was not as informative in finding differences between NT and ASD. In this case, 48 IU is potentially making ASD closer to NT for this network. Next to the feature name is the number that helps measure the changes between treatments. 0 9 means that a particular network was ranked 0 in placebo (most important feature for the placebo), and it moved to the 9^th^ most important feature in the 48 IU condition (i.e., the difference between ASD and NT got much smaller). The final number indicates the overall decrease in the global SHAP vale for this feature. **b** The same description as above applies to the 24 IU IN-OXT condition. **c** The same description as above applies for 8 IU IN-OXT condition.

We conducted a separate group ICA analysis that included all four sessions of ASD (*n* = 30 × 4 doses). Seventeen ICA-derived brain functional networks were identified as not corrupted by motion and other fMRI artifacts (Table S2, Fig. S3, S4 and S5). Among these networks, there was one reward network (ventral striatum and the nucleus accumbens (IC5)), three salience/empathy networks (anterior insula, anterior cingulate cortex (IC18), visual cortex (IC9, IC21)), and two default networks (vmPFC (IC16), and PCC (IC20)). We tracked changes in rsFC between a particular dose of IN-OXT and placebo for ASD (in the direction of PL minus IN-OXT dose) and then compared ASD (red dots) to NT (blue dots). Dots that are to the right of the y-axis signify that the SHAP value in PL is higher than with OT treatment, which means that the importance of this feature that contributes to the difference between ASD and NT is shrinking. In other terms, the treatment is helping to normalize the connectivity between that network in ASD. Qualitative comparison of the effects of the highest dose of IN-OXT (48 IU) to placebo revealed that (1) hyperconnectivity between reward and sensorimotor cortex reduced in ranking and contribution from rank 0 to rank 9 to ASD diagnosis (Fig. 3a and S6); (2) hyperconnectivity between reward and theory of mind areas went from rank 5 of importance with IN-PL to rank 4 with 48 IU of IN-OXT. The hypoconnectivity between salience/empathy and visual cortex reduced in importance from rank 6 with IN-PL to rank 22 with 48 IU of IN-OXT. Thus, the highest dose of IN-OXT administered in the present study reduced connectivity deficits differentiating ASD from NT.

The dose of 24 IU also reduced the ranking of importance of the hyperconnectivity between reward and sensorimotor and theory of mind networks (from rank 0 to rank 4) but did not affect the functional connectivity between the salience/empathy network and visual areas (Fig. 3b). Qualitatively similar results to the middle dose were found with the smaller 8 IU dose (Fig. 3c).

Figure S5 shows the feature importance of the connectivity between two networks of interest for each dose separately to visualize the rank change.

**Fig. 4:**
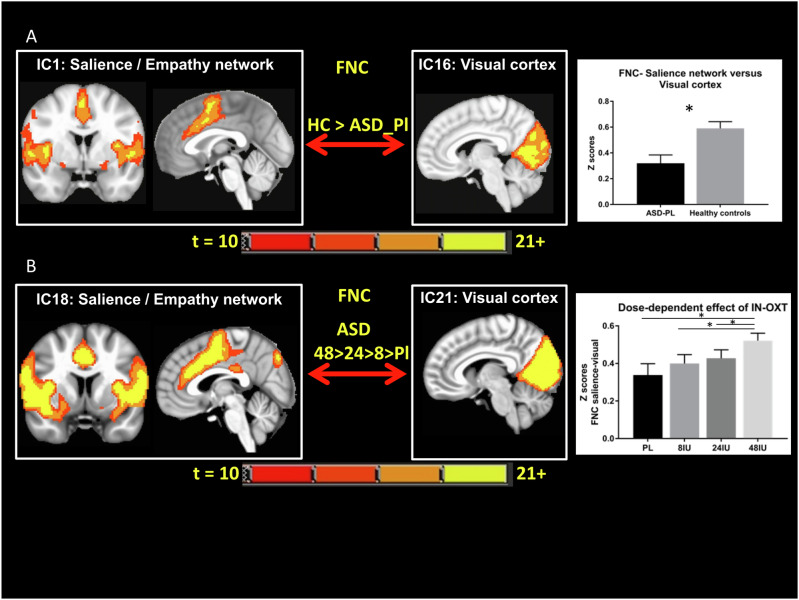
Dose-dependent effects of IN-OXT on rescuing deficits in rsFC between salience/empathy and visual networks. **A** Differences in rsFC between the salience/empathy network and visual cortex between ASD receiving placebo and NT controls. ASD subjects receiving 0 IU IN-OXT (placebo) have hypoconnectivity between these networks compared to NT with IN-PL. **B** Dose-dependent effects of IN-OXT on rsFC between salience/empathy network and visual cortex with the highest dose enhancing this rsFC.

Classical parametric statistical analysis on networks of interest revealed that IN-OXT enhanced the functional connectivity between the salience/empathy network (ACC, anterior insula) and the visual cortex in a dose-dependent manner (Fig. 4) (*F*(3.75) = 4.017, *p* = 0.01, eta square = 0.14). Post-hoc tests using Bonferroni corrections showed that the main effect of dose was driven by the effect of the highest dose (48 IU) of IN-OXT on brain function as compared to placebo (*p* < 0.005).

### DNA methylation of *OXTR* and clinical symptoms mediate the effects of IN-OXT doses on rsFC in ASD

We focused the analyses on the three main networks of interest that consist of the rsFC between (1) salience/empathy and visual cortex, (2) reward and sensorimotor, and (3) reward and theory of mind network. First mixed effects model showed a significant interaction between DNA methylation of −1121 to −1119 site of MT2 area (CpG5.6) and the middle dose of IN-OXT (24 IU) for the salience/empathy and visual cortex connectivity (*t* = −2.66, *p* = 0.009). There was no effect of age (t = −1.1, *p* > 0.28). The 24 IU dose of IN-OXT enhances the rsFC between these networks only in ASD individuals with lower *OXTR* DNA methylation of −1121 to −1119 site of MT2 area (CpG5.6). No interaction was found with 8 IU nor 48 IU and no interaction with IN-OXT doses with regards to rsFC between reward and sensorimotor or theory of mind areas. The second mixed model revealed that ADOS calibrated severity (CS) total scores interacted significantly with middle dose of IN-OXT (24 IU) with regards to the hyperconnectivity between reward and sensorimotor cortex (*t* = 2.71, *p* = 0.024, corrected for the 3 networks analyzed). In other words, the middle dose of IN-OXT (24 IU) reduced hyperconnectivity between the reward and sensorimotor areas in ASD individuals with less severe symptoms (based on ADOS CS scores). There was no effect of age (*t* = −1.43, *p* > 0.164). Exploratory analyses revealed that these effects were mainly driven by the severity of social affect symptoms (*t* = 2.69, *p* = 0.008) and not by repetitive behaviors (*t* = 1.943, *p* = 0.06).

### Effects of IN-OXT doses on peripheral levels of oxytocin in ASD

We found a dose-dependent effect of IN-OXT on plasma levels of oxytocin in ASD individuals (F(3,79) = 20.45, p < 0.001, eta square = 0.44) (see Table S3). Post-hoc analysis revealed that 48 IU enhanced plasma oxytocin levels significantly more than 8 IU (*p* < 0.001) and IN-placebo (*p* < 0.001). A 24 IU and 8 IU intake enhanced plasma oxytocin levels significantly more than IN-placebo (*p* < 0.001, *p* < 0.04, respectively). The elevation of plasma oxytocin levels (post-pre) following 48 IU administration correlated significantly with the effect of the high dose on rsFC between salience networks (*r* = 0.552, *p* = 0.006). We chose to look at this correlation specifically given that it’s the main connectivity showing significant dose-dependent effect of IN-OXT in the previous section (Fig. 4). Plasma level elevation (post-pre) following other doses inhalation did not correlate with the effect of the other doses on rsFC between networks of interest (*p* > 0.05).

## Discussion

**Fig. 5:**
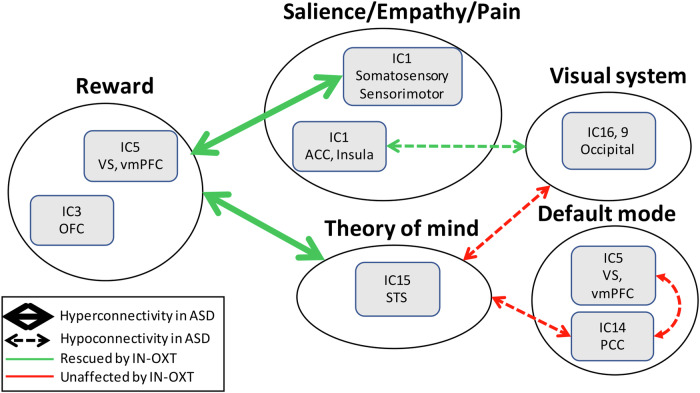
Summary figure illustrating alterations in ASD and effects of IN-OXT on rsFC in ASD. Hyperconnectivity is represented by thick arrows and green color represents deficits that are rescued with IN-OXT. Hypoconnectivity is represented in thin arrows and red color represents deficits that are not rescued by IN-OXT. Hyperconnectivity between the reward and salience and ToM networks is rescued by IN-OXT. Hypoconnectivity between visual and salience networks is rescued by IN-OXT. Hypoconnectivity between ToM and visual and default networks is not rescued by IN-OXT.

We used random forest analysis to classify individuals as ASD or NT as a function of brain connectivity, and we calculated SHAP values to rank the functional networks in the order of importance of prediction of the differences between ASD and NT. Both groups received placebo. We then investigated the effects of varying doses of IN-OXT (0 IU, 8 IU, 24 IU, 48 IU) on resting state functional connectivity between brain networks involved in salience, empathy, and reward in ASD. These analyses revealed that the hyperconnectivity between reward and sensorimotor/somatosensory networks is among the strongest predictors of ASD deficits. Hyperconnectivity between reward and theory of mind networks, and hypoconnectivity between the salience/empathy and visual network in ASD are also among the strong contributors to the differences in brain connectivity between ASD and NT (Fig. 5). Simple statistical analysis confirmed the above findings and revealed deficits in resting-state functional connectivity between the default network and the theory of mind network in ASD, which is in line with previously reported results [18].

The combination of hypo and hyperconnectivity deficits in ASD found in the study is also in line with other findings in the literature [19]. Hyperconnectivity was previously observed in subcortical and striatal areas [20], whereas hypoconnectivity dominated cortico-cortical functional networks [18]. Hyperconnectivity between striatal areas (NAcc) and cortical areas (such as the prefrontal cortex) has been documented in males with ASD and has been associated with a greater number of ASD-associated variants in the *OXTR* gene [21]. The hypoconnectivity between areas involved in salience and empathy processes (ACC and insula) [22] and visual cortex may be related to known empathy deficits in ASD [23].

We also showed that IN-OXT altered the predictability of ASD diagnosis by reducing the deficits that were found initially in rsFC. Higher doses of IN-OXT (48 IU and 24 IU) reduced the hyperconnectivity between reward and sensorimotor and theory of mind networks in ASD. Rescuing these deficits may lead to a reduction in severity of symptoms (repetitive behaviors or sensory sensitivity). Interestingly, middle dose of IN-OXT (24 IU) administered in the present study reduced hyperconnectivity between reward and sensorimotor networks in adults with ASD who have less severe ADOS CS scores. *This suggests that screening for social deficits in ASD prior to clinical trials involving oxytocin is likely to be beneficial for achieving optimal outcomes*.

We found that IN-OXT had a dose-dependent effect on rsFC among anterior insula, ACC and visual cortex (Fg. 5). IN-OXT significantly enhanced the functional connectivity of empathy or salience networks that were found to be deficient in these individuals when they took IN-PL. Interestingly, while the highest dose of IN-OXT (48 IU) enhanced rsFC between these networks in all ASD subjects, 24 IU had stronger effects on rsFC between ACC/insula and visual cortex in ASD subjects with lower DNA methylation of the *OXTR* at −1121 to −1119 site of interest of MT2 area (CpG5.6) that was previously found to be linked to ASD deficits in terms of social responsiveness and in terms of hyperconnectivity between ventral striatum and cortical areas [12]. Therefore, lower levels of methylation can be associated with more normative brain function and more tangible effects of drug on brain function.

*Middle doses of IN-OXT might have more beneficial effects on brain function in adults with ASD who have smaller deficits in the oxytocin receptor expression. Screening for DNA methylation of OXTR prior to oxytocin trials may optimize treatment effects on social and empathy deficits, representing a potential precision medicine approach* [24].

These epigenetic factors related to *OXTR* gene that are moderating IN-OXT’s effects in ASD are in line with previous genetic associations between *OXTR* gene and ASD [25, 26], and between genetic polymorphisms or *SNPs* in *OXTR* and responsiveness to long-term IN-OXT intake in ASD [27]. More specifically, in line with our findings, research has found that daily higher doses of IN-OXT (>21IU) are more effective than lower doses in treating ASD symptoms as measured by CGI-I scores [27]. When dosage was lower, stronger improvement was observed in CGI-I scores for selective subtype of participants with T-allele at rs6791619 [27]. This corroborates our findings as middle doses of IN-OXT modulated brain function in patients who had lower DNA methylation of *OXTR*. Therefore, genetic, and epigenetic factors can predict efficacy of IN-OXT treatment in ASD.

IN-OXT had notable effects on rsFC between key networks of interest previously found to be deficient in ASD. *Our findings suggest that higher doses of IN-OXT may be necessary to achieve a better impact on altering empathy-related mechanisms. Less severe symptomatology of ASD and smaller deficits in OXTR might be good biomarkers for identifying individuals likely to exhibit optimal targeted effects of the drug*. We also found a target engagement effect of intranasal doses on blood oxytocin peptide levels. Higher levels of oxytocin in plasma correlated with oxytocin’s effects on rsFC between empathy and salience networks in ASD.

The linear dose-response curve we observe in this study might seem to contradict the previously inverted U shape dose-response curve findings on oxytocin [28–30]. However, some of these studies explore acute effects of IN-OXT in the context of fear and anxiety (which is not the case in our study) [28] and given that high doses of IN-OXT can enhance functional connectivity in networks involved in salience (based on our findings), lower doses may be better able to reduce anxiety and fear. Other studies used IN-OXT repeatedly in long-term trials and found that lower doses are more efficient which could be related to the problem of saturating receptors and binding to vasopressin.

These brain-based oxytocin dose-dependent findings can be informative for future treatment trials. Given that hyperconnectivity between reward and sensorimotor cortices is usually linked to sensory problems and repetitive behaviors in ASD [31], we speculate that a subtype of ASD that has predominantly these issues can benefit from high doses of IN-OXT and that lower doses can be given to those who have fewer social deficits. In the context of social communication and empathy, higher doses of IN-OXT are more likely to improve these functions. Even though anxiety and negative valence were not directly or indirectly (brain networks) addressed here, it is possible that lower doses of IN-OXT can be more beneficial for patients who are suffering from high neuroticism as higher doses can enhance social salience and therefore not help with anxiety reduction. This can explain in part the lack of positive findings in previous clinical trials using high doses of IN-OXT daily for an extended period in children with ASD to test its effects on social withdrawal which is more related to social anxiety and depression [10], and in trials using lower doses of IN-OXT to study its effects on social communication and responsiveness [32, 33].

By targeting these selective networks and specific deficits (hypoconnectivity between salience and visual networks and hyperconnectivity between reward and sensorimotor networks) in ASD, future research can conduct hypothesis-based research (not exploratory) to correlate these deficits in functional connectivity to specific subtypes of ASD characterized by behavioral deficits in domains of social cognition, sensory function, or empathy. Future clinical trials can also target these functional networks or ASD subtypes that have these specific deficits when administering oxytocin or other drugs that have similar mechanisms of action.

Limitations in the study include a lack of behavioral measures that can predict IN-OXT dose-dependent effects in ASD in addition to its effects on brain function. Future studies assessing empathic abilities and sensory skills in ASD subjects will be important to correlate with rsFC. Another limitation consists of exploring differences between NT and ASD in terms of rsFC not at baseline but after placebo intake (for both groups). NT came for only one visit of placebo and ASD subjects came for several visits, including one with placebo. However, our results in terms of deficits in ASD are in line with previous literature, which makes the results less likely to have been biased by placebo intake. We did not have a comparison control group to compare IN-OXT effects on brain function between ASD and NT which is another limitation. Most of subjects with ASD were on psychotropic medication during the trial which could have affected brain function results. However, this limitation is minimized by the nature of our within-subject study design.

Machine learning approaches used in the study led to findings that are consistent with previous literature (in terms of hyper and hypoconnectivity deficits in ASD and in terms of effects of the drug on salience network for instance). However, applying these methods in larger clinical trials (greater sample size) in a wide sample of ASD (including women with ASD, different levels of support needs, and others) and targeting behavior and brain function with different doses of IN-OXT would be key to add consistency and validity to our methods and findings.

Additionally, our sample size is modest, and results are specific to males with preserved cognitive and language capacities. We included adults with ASD and controls as it is mainly a mechanistic study, and it involves recurring neuroimaging scans which can be challenging for children to perform. Replications in larger and broader samples including women and additional demographic groups are needed. Future studies involving social tasks and naturalistic settings can inform us about targeted effects of IN-OXT and whether they are different from its effects on intrinsic functional connectivity found in this study. Even though we aimed to use a personalized medicine approach to study how IN-OXT brain-based targeted effects can be moderated by epigenetic and clinical factors, additional factors not covered here may also contribute to the individual differences in responses to the drug. Using multidimensional (social function, sensory processing, etc.) and multimodal methodologies (behavior, brain imaging, genetics, etc.) and investigating how subtypes of ASD characterized by their unique features vary in their responses to IN-OXT treatment can yield a more nuanced personalized approach.

*In summary*, higher doses of IN-OXT might be necessary to achieve optimal outcomes on salience/empathy mechanisms during clinical trials using acute treatments or before behavioral therapies. Optimization can also be achieved by delivering intermittent higher doses (to avoid receptor saturation) [34] and targeting outcome measures related to attention and emotional responsiveness. Our study offers insights into personalized treatment approaches and prospective practical implications for real-world clinical treatment trials. Our brain-based limited observations emphasize that individuals with ASD with less severe social deficits might benefit from moderate doses of IN-OXT. We also speculate that individuals with ASD with low *OXTR* DNA methylation in the specific site mentioned above and with predominant difficulties in affective empathy and attention to social cues (or social salience) might benefit from moderate doses of IN-OXT. Theory of mind tasks and cognitive tasks are less likely to be affected by OXT treatment. Our study also shows the value of applying machine learning techniques and data-driven unbiased predictions of factors that contribute to ASD deficits and drug treatment effects. Future research incorporating these types of analyses might lead to more consistency across the literature.

## Supplementary information

Supplemental Material

## Data Availability

Datasets will be available to readers and will be presented in a supporting file.
