# Supplementary material for: Random forest and Shapley Additive exPlanations predict oxytocin targeted effects on brain functional networks involved in salience and sensorimotor processing, in a randomized clinical trial in autism
Source: Neuropsychopharmacology. 2025 Apr 2;50(9):1385–94. doi: 10.1038/s41386-025-02095-2 (PMC12260019; doi:10.1038/s41386-025-02095-2)
Supplement: Supplementary file 1 — Supplemental Material [file 41386_2025_2095_MOESM1_ESM.pdf]

Materials and Methods

Participants

We recruited 50 adult subjects with ASD from the Emory Autism Center as part of the Emory University IRB protocol #63466 between 2017 and 2019. We originally conducted power analysis in the main IRB protocol (G power 3.1 software) and found that a sample size of 50 subjects with ASD receiving 4 different treatments is needed (95% power) to reliably detect small effect sizes. However, we expected to have larger effect sizes for the brain effects of IN-OXT.

Of these, 40 participants completed a series of clinical and behavioral tasks. Some behavioral tasks are not reported here in the manuscript and will be published in separate manuscripts. Out of these 40 participants, 32 adult men with ASD (between the ages of 18 and 45) were recruited to participate in the brain imaging study presented here (The Autism Oxytocin Brain project (AOB), Emory IRB #9455). Around half of these remaining 8 families were not available or not reachable, while the other half were not willing to participate in a drug trial. Families and patients were invited to visit the Emory University Hospital in Atlanta. The visits occurred at the Georgia Clinical and Translational Science Alliance Clinical Research Center (GCRCs). One participant withdrew from the imaging study because of anxiety symptoms inside the MRI, resulting in an N=31. One patient's data were excluded from the fMRI analysis because of technical problems with the acquisition of data (N=30 for data analysis). ASD diagnosis was confirmed with the gold-standard tools of Autism Diagnostic Observation Schedule (ADOS) and Autism Diagnostic Interview (ADI). Parents also completed the Social Responsiveness Scale (SRS-second edition). Eligibility inclusion criteria consisted of being between 18 and 45 years old, intellectual quotient >70 and corrected to normal vision (see supplemental material). Exclusion criteria consisted of recent occurrence of seizures (within the past 5 years), brain damage or head trauma, alcoholism or

substance abuse, presence of a severe medical problem, cardiovascular disease, current and untreated asthma and migraines, claustrophobia, intellectual disabilities, pacemakers, cochlear implants, surgical clips, or metal fragments.

We also recruited 19 neurotypical individuals (NT) who were willing to participate in the clinical trial (men, matched for age and IQ with our ASD subjects). Two participants withdrew due to lack of interest, leading to 17 controls participating in the trial.

Experimental procedure

Prior to drug administration, temperature, heart rate, and blood pressure were measured by the nurse. If the heart rate was above 100 bpm, or the blood pressure was above 140/90, vitals were re-measured. If levels remained elevated after three successive recordings in both limbs measured at least 5 minutes apart, the study physician was notified and asked to advise. Vitals were also administered 15 minutes after spray intake. If temperature had increased or decreased by more than 2 degrees, heart rate was above 100 bmp, or blood pressure was above 140/90, the vitals were remeasured, and adverse events were reported.

We selected 5 minutes blood withdrawal instead of 10 minutes after spray intake because of logistics reasons as the inhalation of spray can take a significant time with ASD subjects. We therefore calculated that the blood draw is taken around 10 min after the beginning of spray intake. We also had to schedule vitals 15 min after spray intake and therefore we want to leave some time to rest following blood collection and before vitals are taken.

Intranasal OXT administration

For the intranasal administration, subjects were instructed to clear their nose before administration. The nurse primed the bottle first by spraying in the air. She then instructed the

subject to sit with the head upright with slight backward tilt. The subject closed one nostril with one finger while the nurse administered the spray to the other nostril continuously.

The placebo solution was compounded by the local Emory Hospital pharmacy and contained all the inactive ingredients without oxytocin. The local pharmacy was also charged with dose preparation where they rebottled the spray individually into sterile empty bottles, based on a pre-planned randomized double-blind design.

Twelve puffs were delivered for all doses across all visits. For each dose during each visit, we used three bottles of 1ml with 6, 4, and 2 puffs, respectively, that contained Syntocinon or/and placebo based on the dose. For 8 IU, there was one bottle with the drug (2 puffs) and 2 bottles of placebo. For 24 IU, there were two bottles of the drug (2 and 4 puffs) and one bottle with placebo. For 48 IU, all three bottles contained the drug.

All NT participants received 12 puffs of IN-PL

fMRI acquisition and pre-processing

In addition a 5-measurement EPI scan with the same parameters as the rsfMRI scan but with reversed phase encoding was acquired to assist in distortion correction [1]. A whole-brain 3D T1-weighted MPRAGE sequence (FOV = 256 mm; TR/TI/TE/FA = 2530 ms/1100 ms/2.96 ms/7°; 1 mm x 1 mm x 1 mm resolution) provided anatomic detail. Foam padding was provided to minimize subject-motion during the scans.

The rsfMRI voxel time-series were corrected for magnetic susceptibility induced geometric distortions [1], temporally shifted to account for differences in slice acquisition times, 3D volume registered to a base volume to account for global rigid motion, and spatially smoothed with an isotropic Gaussian filter (FWHM = 5 mm). The resulting images were detrended of motion-related

and physiological artifacts using the ICA-AROMA technique [2]. The artifact-corrected rsfMRI time-series were aligned to the MNI152 template by applying the warp (image transformation) computed from the registration of unsmoothed EPI time-series to MNI152 template; and resampled to 3mm X 3mm X 3mm voxel resolution. In order to compute the warp of the fMRI time-series from MRI scanner space to the MNI152 space, the unsmoothed the base volume of the 3D volume registration step mentioned above was co-registered to the T1-weighted high-resolution anatomic scan using the affine boundary based registration algorithm [3], and spatially normalized to the MNI152 template with the warp computed from alignment of the high-resolution 3D anatomical scan to the MNI152 template using a nonlinear registration algorithm. The matrix of transformation from EPI to T1-weighted high-resolution anatomic, and the nonlinear warp computed to align the T1-weighted high-resolution anatomic to MNI152 template were combined to form the EPI-to-MNI152 warp and applied to the output of the ICA-AROMA through FSL's *applywarp* program. There were 160 measurements in each rsfMRI scan.

rsfMRI data analysis

Group Independent Component Analysis (ICA) was performed, and functional network connectivity (FNC) was analyzed. Group spatial ICA (GICA), a method for making group inferences from functional MRI data using independent component analysis, was performed on temporally concatenated data of the whole group [4] using the GIFT software [5]. Initial GICA was performed with automatic estimation of the number of independent components (ICs) using the minimum description length (MDL) criteria. The ICASSO utility of the GIFT software with random initialization and bootstrapping was used to ensure the stability/reliability of the ICA [5]. Whole-brain maps of strength of the artifact-free ICs (expressed as z-scores), as well as

corresponding IC time-series, for each subject were generated by back-reconstruction of the group aggregate mixing matrix [4, 5]. We used the Neurosynth database of brain statistical maps [6] to identify the different networks based on 1000 data points of meta-analysis studies.

Random forest analysis.

Briefly, a decision tree is a supervised model that takes data of the form $(\boldsymbol{X}, \boldsymbol{Y})$ where $\boldsymbol{X}$ is the $n \; x \; p$ matrix of observations and features, and $\boldsymbol{Y}$ is the $n \; x \; 1$ vector of targets. In this case, $y_i \in \{ASD, NT\}$, i.e. the targets are the class labels of ASD or NT. At each level of the tree a feature $\boldsymbol{x_j} \in \boldsymbol{X}$ and a split point s are chosen such that observations filtered based on the chosen split point have maximally homogenous targets.

$$R_1(j, s) = \{X \mid X_j \leq s\}, \;\; R_2(j, s) = \{X \mid X_j > s\}$$

R_1 and R_2 are the resulting subsets of $\boldsymbol{X}$ after a feature $j$and split point s have been chosen. To compensate for the substantial variance, an ensemble supervised learning technique trains many decision trees and averages their output to smooth the model and reduce variance. Each of these trees is given a subset of the training data, and instead of considering all the features at each level, a random subset of the features $m \leq n$ is used.

Oxytocin peptide measurement in plasma

Briefly, plasma samples (1 ml per sample) were extracted using an Oasis PRiME HLB column with 60 mg sorbent (Waters Inc.) and evaporated at room temperature using compressed nitrogen. Each evaporated sample was reconstituted in 250 µl of assay buffer before OT measurement to provide sufficient sample volume to run each sample in duplicate wells (100 µl per well). The samples were extracted and assayed using a microplate reader (Synergy HTX,

Agilent Technologies, Inc.) in a 96-well format according to the manufacturer's instructions. The intraassay coefficient of variation was 14.8%.

References

1- Glasser MF, Sotiropoulos SN, Wilson JA, Coalson TS, Fischl B, Andersson JL, et al. The minimal preprocessing pipelines for the Human Connectome Project. Neuroimage. 2013;80:105-24.

2- Pruim RHR, Mennes M, van Rooij D, Llera A, Buitelaar JK, Beckmann CF. ICA-AROMA: A robust ICA-based strategy for removing motion artifacts from fMRI data. Neuroimage. 2015;112:267-277.

3- Greve DN, Fischl B. Accurate and robust brain image alignment using boundary-based registration. Neuroimage. 2009;48:63-72.

4- Calhoun VD, Adali T, Pearlson GD, Pekar JJ. A method for making group inferences from functional MRI data using independent component analysis. Hum Brain Mapp. 2001;14:140-51.

5- Calhoun VD, Liu J, Adali T. A review of group ICA for fMRI data and ICA for joint inference of imaging, genetic, and ERP data. Neuroimage. 2009;45:S163-72.

6- Yarkoni T, Poldrack RA, Nichols TE, Van Essen DC, Wager TD. Large-scale automated synthesis of human functional neuroimaging data. Nat Methods. 2011;8:665-70.

Figure legends.

S1 and S2. These figures show all the IC networks that are above noise based on the independent component analysis in terms of differences between ASD who took placebo and NT who took placebo. Differences were found in the salience network, reward network, default network, theory of mind network, visual network, sensorimotor network, and fronto-parietal networks.

S3. ROC curve showing 90% accuracy in predicting ASD diagnosis in the random forest model

S4 and S5. These figures show all the IC networks that are above noise when comparing IN-OXT doses. Salience network, reward network, visual network, default and theory of mind networks, fronto-parietal networks, sensorimotor networks and cerebellum and brainstem show significant results above noise.

S6. SHAP analysis show the main contributors in terms of functional networks in predicting each dose of IN-OXT in comparison to placebo.

Table S1 and S2. These tables show all the IC networks corresponding brain function domains based on neurosynth analysis.

Table S3. This table shows plasma oxytocin levels (pg.ml) before and after drug administration

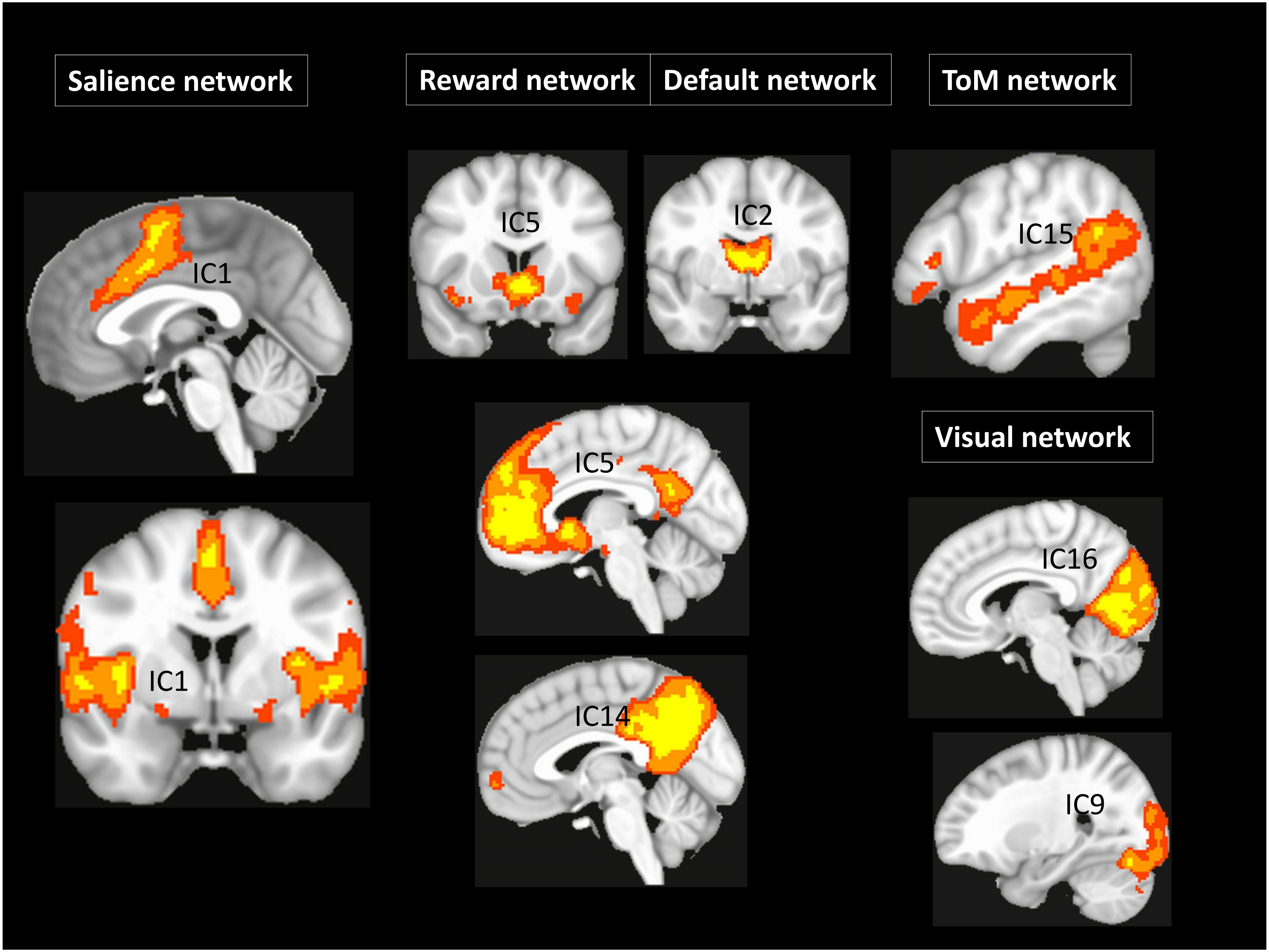

Salience network
IC1
IC1
Reward network
IC5
IC5
Default network
IC2
IC14
ToM network
IC15
Visual network
IC16
IC9

Figure S1

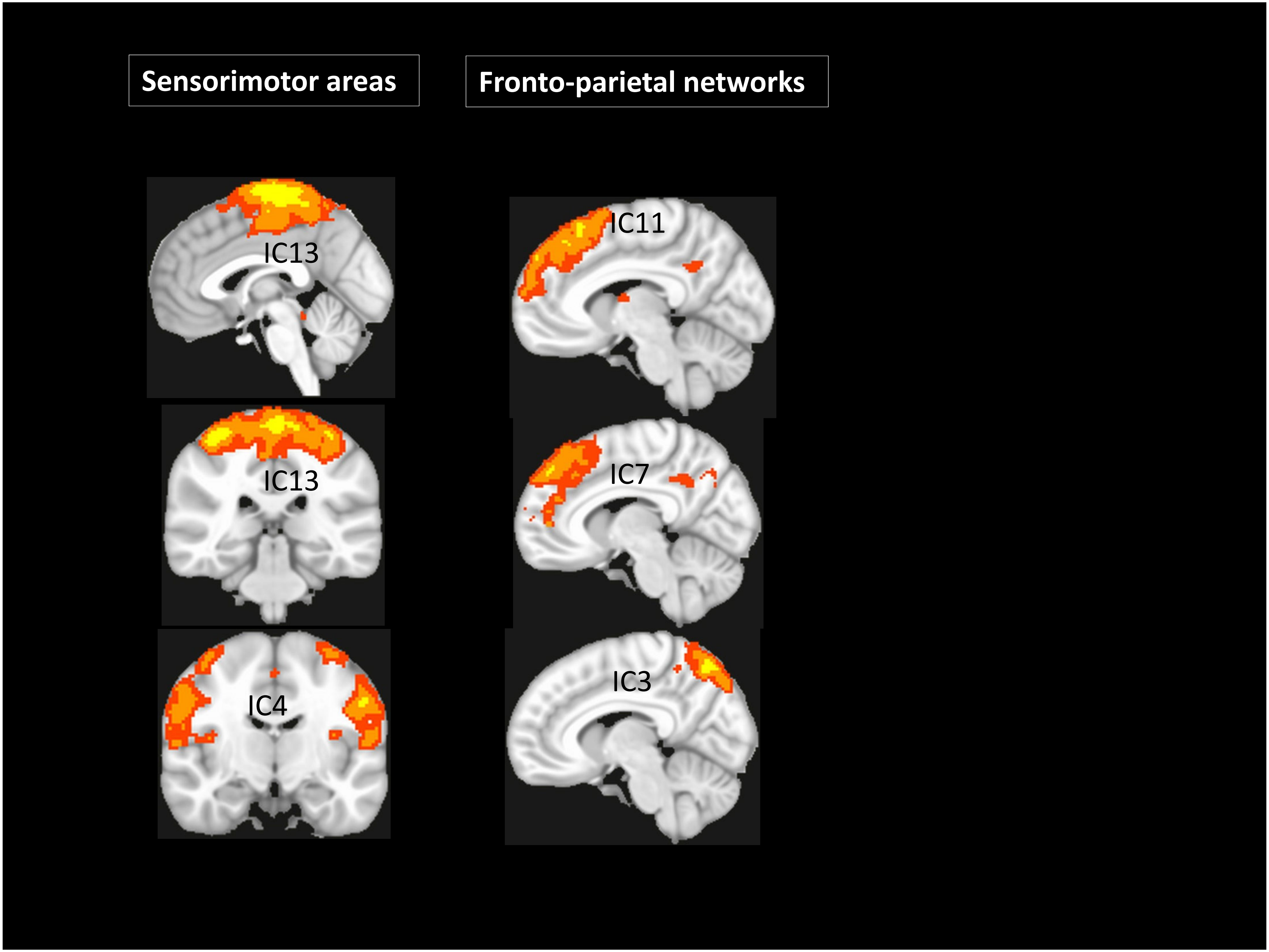
Sensorimotor areas
Fronto-parietal networks
IC13
IC13
IC4
IC11
IC7
IC3

Figure S2

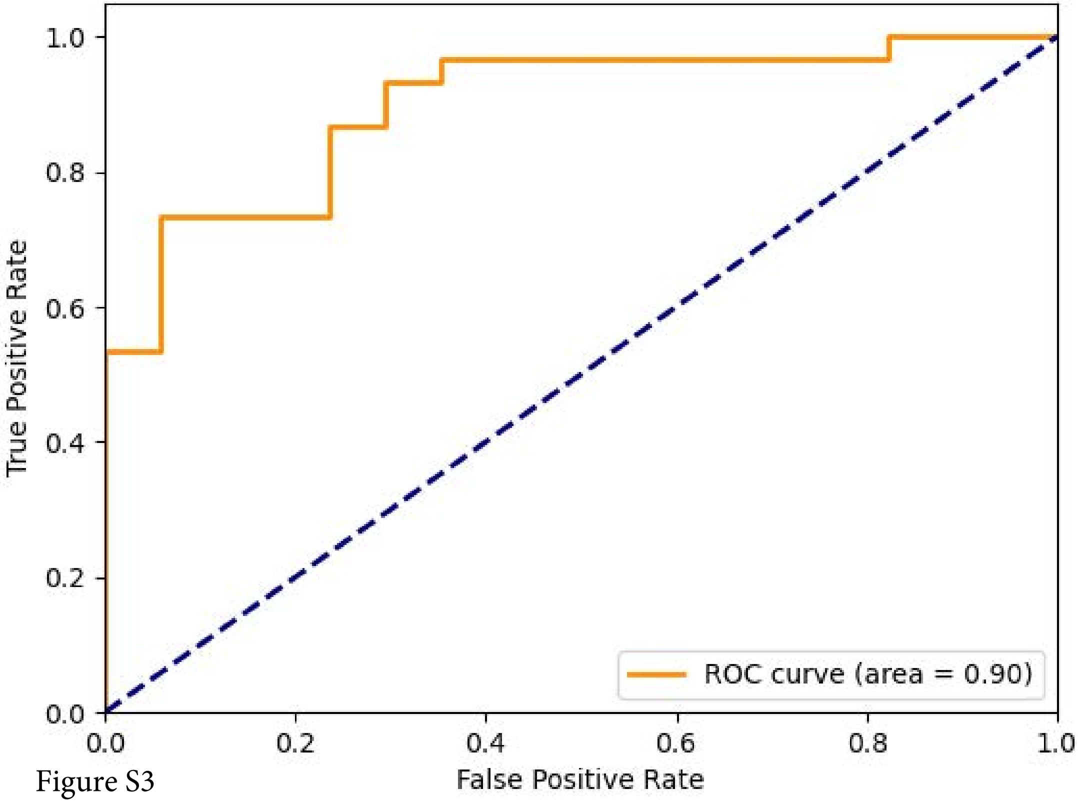
1.0
0.8
0.6
0.4
0.2
0.0
True Positive Rate
0.0
0.2
0.4
0.6
0.8
1.0
False Positive Rate
ROC curve (area = 0.90)

Figure S3

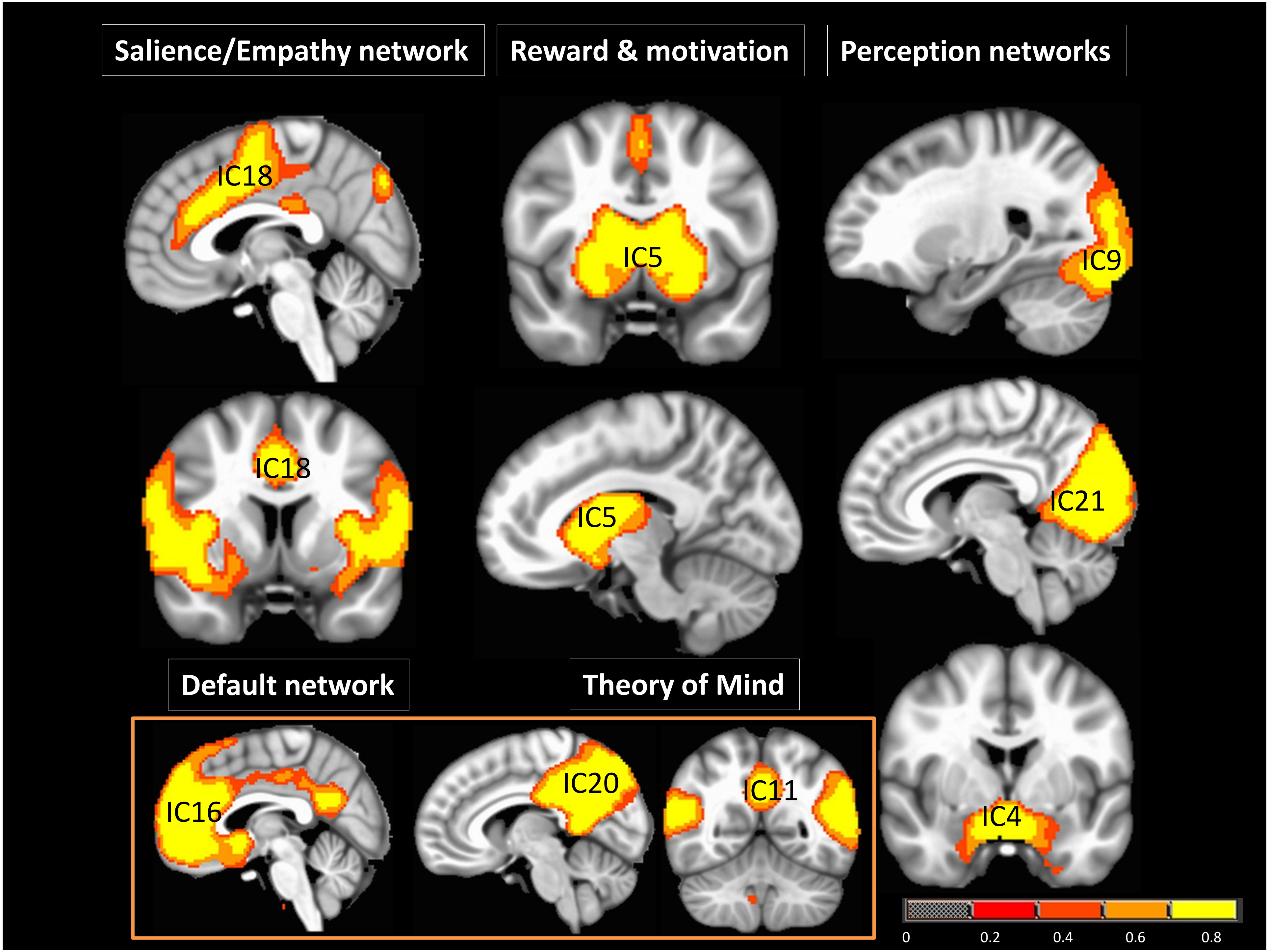
Salience/Empathy network
Reward & motivation
Perception networks
IC18
IC5
IC9
IC18
IC5
IC21
Default network
Theory of Mind
IC16
IC20
IC11
IC4
0
0.2
0.4
0.6
0.8

Figure S4

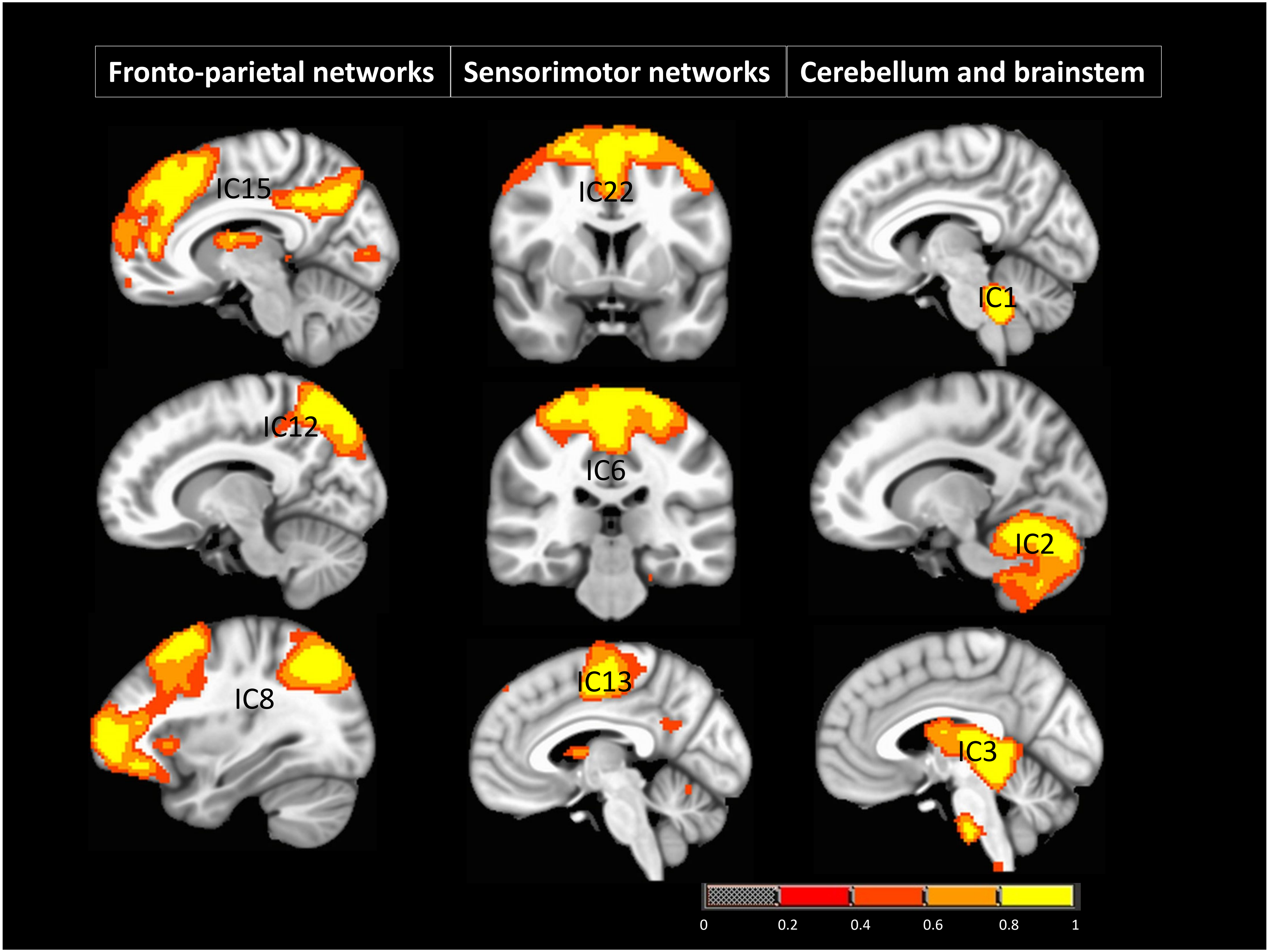
Fronto-parietal networks
Sensorimotor networks
Cerebellum and brainstem
IC15
IC22
IC1
IC12
IC6
IC2
IC8
IC13
IC3
0
0.2
0.4
0.6
0.8
1

Figure S5

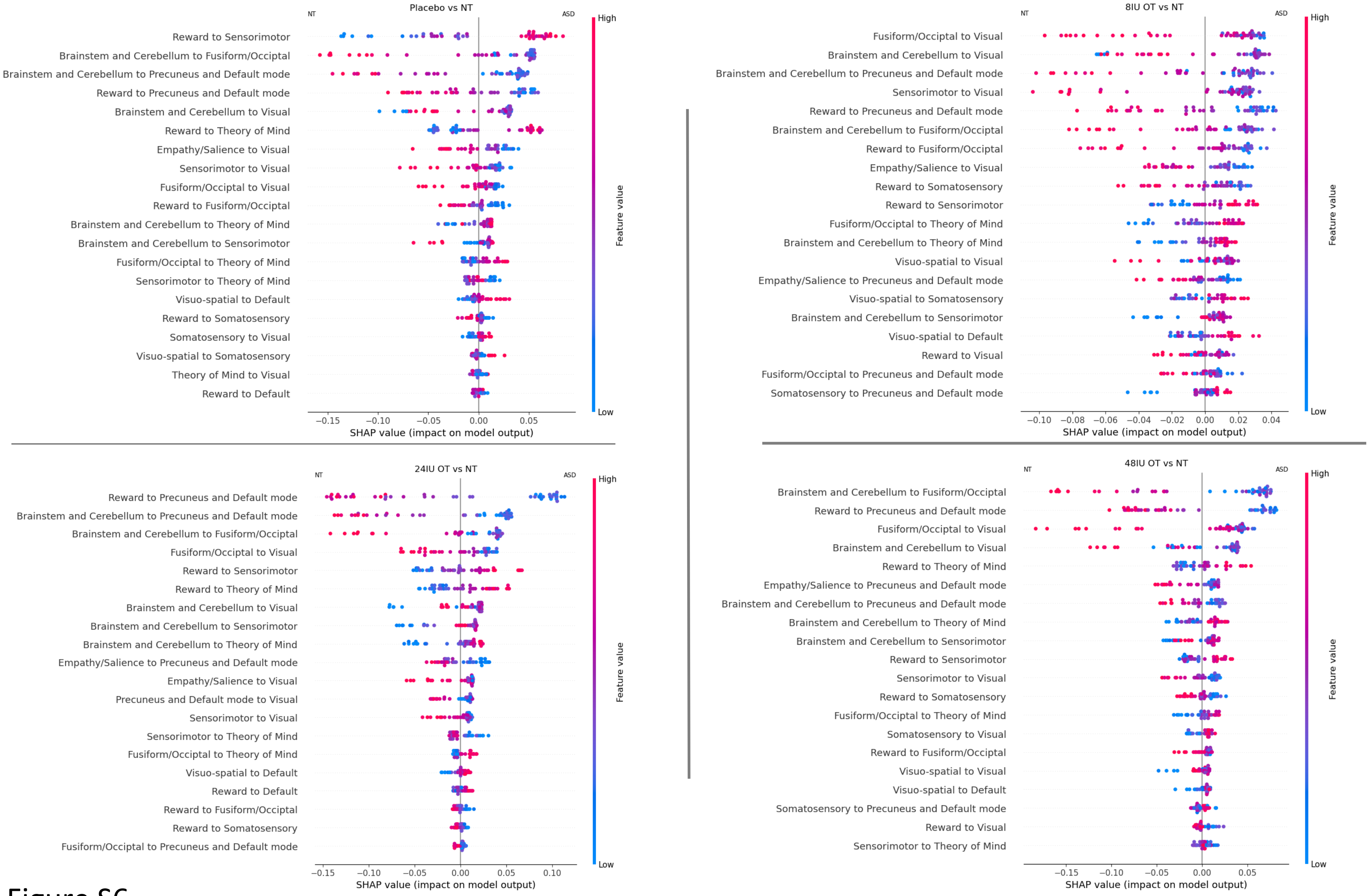

Placebo vs NT
NT
ASD
Reward to Sensorimotor
Brainstem and Cerebellum to Fusiform/Occipital
Brainstem and Cerebellum to Precuneus and Default mode
Reward to Precuneus and Default mode
Brainstem and Cerebellum to Visual
Reward to Theory of Mind
Empathy/Salience to Visual
Sensorimotor to Visual
Fusiform/Occipital to Visual
Reward to Fusiform/Occipital
Brainstem and Cerebellum to Theory of Mind
Brainstem and Cerebellum to Sensorimotor
Fusiform/Occipital to Theory of Mind
Sensorimotor to Theory of Mind
Visuo-spatial to Default
Reward to Somatosensory
Somatosensory to Visual
Visuo-spatial to Somatosensory
Theory of Mind to Visual
Reward to Default
−0.15 −0.10 −0.05 0.00 0.05
SHAP value (impact on model output)
High
Feature value
Low
8IU OT vs NT
NT
ASD
Fusiform/Occipital to Visual
Brainstem and Cerebellum to Visual
Brainstem and Cerebellum to Precuneus and Default mode
Sensorimotor to Visual
Reward to Precuneus and Default mode
Brainstem and Cerebellum to Fusiform/Occipital
Reward to Fusiform/Occipital
Empathy/Salience to Visual
Reward to Somatosensory
Reward to Sensorimotor
Fusiform/Occipital to Theory of Mind
Brainstem and Cerebellum to Theory of Mind
Visuo-spatial to Visual
Empathy/Salience to Precuneus and Default mode
Visuo-spatial to Somatosensory
Brainstem and Cerebellum to Sensorimotor
Visuo-spatial to Default
Reward to Visual
Fusiform/Occipital to Precuneus and Default mode
Somatosensory to Precuneus and Default mode
−0.10 −0.08 −0.06 −0.04 −0.02 0.00 0.02 0.04
SHAP value (impact on model output)
High
Feature value
Low
24IU OT vs NT
NT
ASD
Reward to Precuneus and Default mode
Brainstem and Cerebellum to Precuneus and Default mode
Brainstem and Cerebellum to Fusiform/Occipital
Fusiform/Occipital to Visual
Reward to Sensorimotor
Reward to Theory of Mind
Brainstem and Cerebellum to Visual
Brainstem and Cerebellum to Sensorimotor
Brainstem and Cerebellum to Theory of Mind
Empathy/Salience to Precuneus and Default mode
Empathy/Salience to Visual
Precuneus and Default mode to Visual
Sensorimotor to Visual
Sensorimotor to Theory of Mind
Fusiform/Occipital to Theory of Mind
Visuo-spatial to Default
Reward to Default
Reward to Fusiform/Occipital
Reward to Somatosensory
Fusiform/Occipital to Precuneus and Default mode
−0.15 −0.10 −0.05 0.00 0.05 0.10
SHAP value (impact on model output)
High
Feature value
Low
48IU OT vs NT
NT
ASD
Brainstem and Cerebellum to Fusiform/Occipital
Reward to Precuneus and Default mode
Fusiform/Occipital to Visual
Brainstem and Cerebellum to Visual
Reward to Theory of Mind
Empathy/Salience to Precuneus and Default mode
Brainstem and Cerebellum to Precuneus and Default mode
Brainstem and Cerebellum to Theory of Mind
Brainstem and Cerebellum to Sensorimotor
Reward to Sensorimotor
Sensorimotor to Visual
Reward to Somatosensory
Fusiform/Occipital to Theory of Mind
Somatosensory to Visual
Reward to Fusiform/Occipital
Visuo-spatial to Visual
Visuo-spatial to Default
Somatosensory to Precuneus and Default mode
Reward to Visual
Sensorimotor to Theory of Mind
−0.15 −0.10 −0.05 0.00 0.05
SHAP value (impact on model output)
High
Feature value
Low

Figure S6

IC networks	NEUROSYNTH – BRAIN AREAS	NEUROSYNTH – DOMAINS - FUNCTION
IC1	anterior insula, ACC, somatosensory, superior temporal, SMA	Pain, Empathy
IC2	caudate, sub-cortical, basal ganglia, striatum	Reward
IC3	parietal, occipital	Executive function
IC4	sensorimotor, somatosensory	Sensorimotor
IC5	mPFC, cingulate, ACC, PCC, amygdala, ventral striatum, OFC, nucleus accumbens	Social, emotional, reward, default mode
IC6	cerebellum brainstem VTA	Cerebellum
IC9	Occipital, fusiform	Visual
IC11	Inferior frontal, inferior parietal, PFC	Language
IC13	Sensorimotor, SMA, motor cortex	Sensorimotor
IC14	precuneus, PCC	Default
IC15	superior temporal, STS	Theory of mind
IC16	occipital	Visual

Table S1

IC networks	Neurosynth – BRAIN AREAS	NEUROSYNTH – DOMAINS - FUNCTION
IC1	Cerebellum, brainstem	Cerebellum
IC2	Cerebellum	Cerebellum
IC3	Thalamus, periaqueductal grey	Thalamus
IC5	Caudate, ventral striatum	Reward
IC6	Sensorimotor cortex	Sensorimotor
IC8	Parietal and Frontal cortex	Executive function
IC9	Occipital and visual cortex	Visual
IC11	Superior Temporal Sulcus	Theory of mind
IC12	Parietal cortex	Parietal
IC13	SMA, motor cortex	Sensorimotor
IC15	Prefrontal cortex, inferior parietal cortex	Language
IC16	vmPFC, ventral striatum	Default mode
IC18	Anterior insula, ACC	Pain, empathy
IC20	Precuneus, posterior cingulate cortex	Default
IC21	Visual cortex	Visual
IC22	Motor cortex, SMA	Sensorimotor

Table S2

	48 IU pre	48 IU post	24 IU pre	24 IU post	8 IU pre	8 IU post	PL pre	PL post
Average	9.724834821	20.86470089	9.060504464	17.52154464	11.17454107	14.36141071	8.088142857	7.745955357
Standard Error	1.777147931	2.391795164	0.651420783	1.589079042	1.320586511	1.753331349	0.749667408	0.692500168

Table S3: Plasma oxytocin levels (pg/ml) before and after drug administration

Table S3

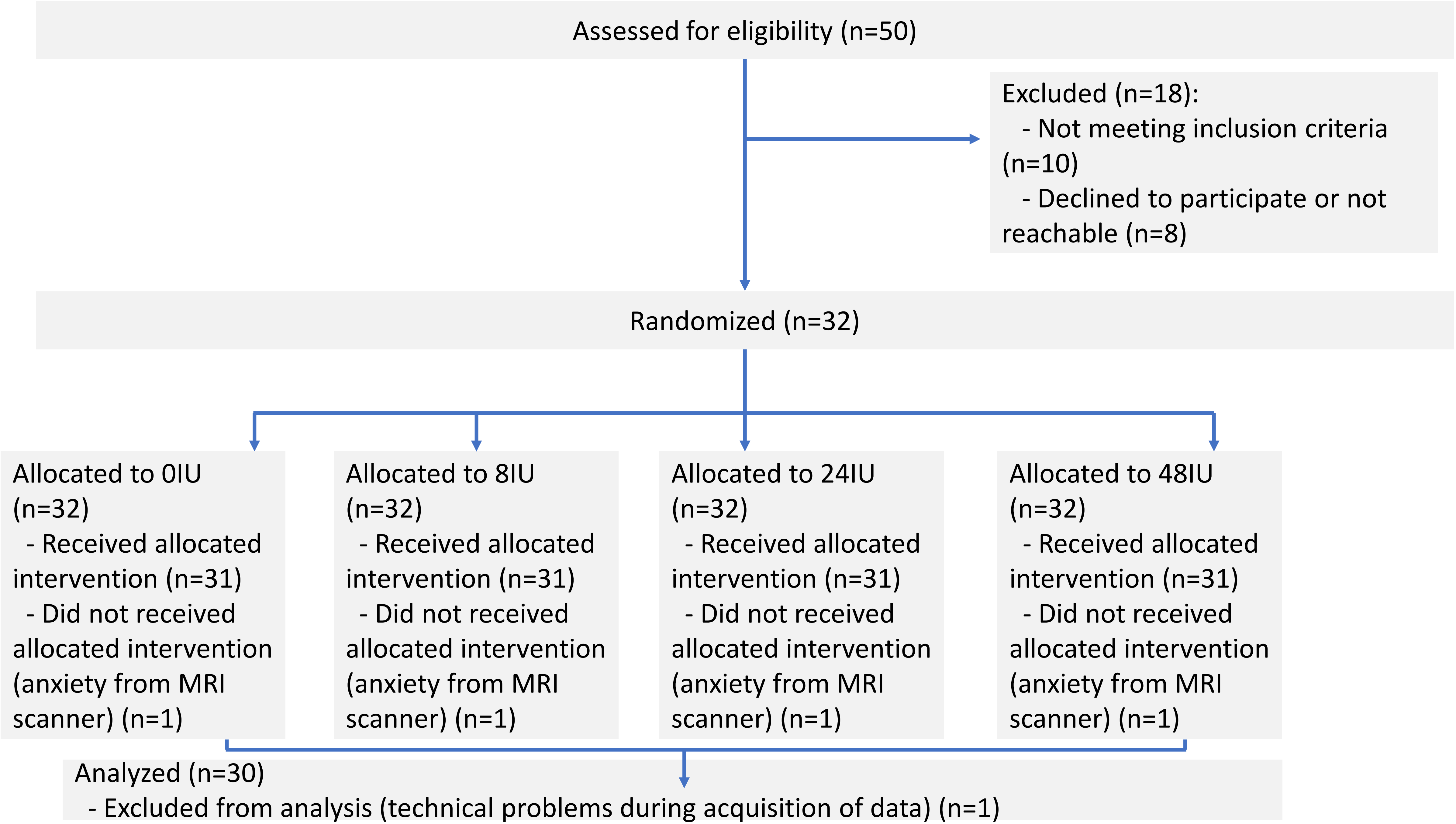
Assessed for eligibility (n=50)
Excluded (n=18):
- Not meeting inclusion criteria (n=10)
- Declined to participate or not reachable (n=8)
Randomized (n=32)
Allocated to 0IU (n=32)
- Received allocated intervention (n=31)
- Did not received allocated intervention (anxiety from MRI scanner) (n=1)
Allocated to 8IU (n=32)
- Received allocated intervention (n=31)
- Did not received allocated intervention (anxiety from MRI scanner) (n=1)
Allocated to 24IU (n=32)
- Received allocated intervention (n=31)
- Did not received allocated intervention (anxiety from MRI scanner) (n=1)
Allocated to 48IU (n=32)
- Received allocated intervention (n=31)
- Did not received allocated intervention (anxiety from MRI scanner) (n=1)
Analyzed (n=30)
- Excluded from analysis (technical problems during acquisition of data) (n=1)
